# Activation Energy and Bipolar Switching Properties for the Co-Sputtering of ITO_X_:SiO_2_ Thin Films on Resistive Random Access Memory Devices

**DOI:** 10.3390/nano13152179

**Published:** 2023-07-26

**Authors:** Kai-Huang Chen, Chien-Min Cheng, Na-Fu Wang, Ming-Cheng Kao

**Affiliations:** 1 Department of Electronic Engineering, Center for Environmental Toxin and Emerging-Contaminant Research, Super Micro Mass Research & Technology Center, Cheng Shiu University, Chengcing Rd., Niaosong District, Kaohsiung 83347, Taiwan; 5977@gcloud.csu.edu.tw (K.-H.C.); k0481@gcloud.csu.edu.tw (N.-F.W.); 2 Department of Electronic Engineering, Southern Taiwan University of Science and Technology, Tainan 710301, Taiwan; 3 Department of Information and Communication Engineering, Chaoyang University of Technology, Taichung 413310, Taiwan

**Keywords:** activation energy, bipolar resistance switching, resistive random access memory, electrical conduction mechanisms, ITO_X_:SiO_2_

## Abstract

Activation energy, bipolar resistance switching behavior, and the electrical conduction transport properties of ITO_X_:SiO_2_ thin film resistive random access memory (RRAM) devices were observed and discussed. The ITO_X_:SiO_2_ thin films were prepared using a co-sputtering deposition method on the TiN/Si substrate. For the RRAM device structure fabrication, an Al/ITO_X_:SiO_2_/TiN/Si structure was prepared by using aluminum for the top electrode and a TiN material for the bottom electrode. In addition, grain growth, defect reduction, and RRAM device performance of the ITO_X_:SiO_2_ thin film for the various oxygen gas flow conditions were observed and described. Based on the *I-V* curve measurements of the RRAM devices, the turn on-off ratio and the bipolar resistance switching properties of the Al/ITO_X_:SiO_2_/TiN/Si RRAM devices in the set and reset states were also obtained. At low operating voltages and high resistance values, the conductance mechanism exhibits hopping conduction mechanisms for set states. Moreover, at high operating voltages, the conductance mechanism behaves as an ohmic conduction current mechanism. Finally, the Al/ITO_X_:SiO_2_/TiN/Si RRAM devices demonstrated memory window properties, bipolar resistance switching behavior, and nonvolatile characteristics for next-generation nonvolatile memory applications.

## 1. Introduction

Recently, nonvolatile resistance random access memory (RRAM) devices have been investigated and studied because of the simple device structure, nonvolatile properties, excellent operation speed, two-state possibility, device size, packing density, and low power loss [1,2,3,4,5,6,7,8,9,10,11,12]. Various nonvolatile memory devices have been widely used for portable electronic systems, such as personal digital assistants (PDA), cell phones, digital cameras, and flash storage devices. The nonvolatile memory properties and the resistance switching properties of the RRAM devices are affected by high current state (LRS) and low current state (HRS) bipolar switching characteristics. Various RRAM device materials, such as chalcogenide oxide material, metal oxide base material, carbide oxide, polymers, and metal doped silicon, have been discovered in the past [1,2,3,4,5,6,7,8,9,10,11,12,13,14,15,16,17,18,19,20].

Metal doped silicon-based oxide thin films have been considerably studied and investigated for applications in nonvolatile resistive random access memory (RRAM) devices; among them are many nonvolatile memory devices such as ferroelectric random access memory (FeRAM), magnetic random access memory (MRAM), and phase change memory (PCM) [1,2,3,4,5,6,7,8,9,10,11,12,13]. The advantages of electronic memory cards, intelligent electronic devices, and related portable electric devices using silicon-based oxide thin films were excellent compatibility with the integrated circuit (IC) process, non-destructive readout properties, low operating voltage, high computing speed, long read/write retention time, and simple structure [19,20,21,22,23,24]. In addition, this metal doped in silicon dioxide material has also been widely studied and applied in modern CMOS device technology [23]. Defects and impurities of the SiO_2_-based RRAM devices are critical to the realization of the resistive switching characteristics and behavior. Indium tin oxide (ITO) materials have been widely used as transparent electrode materials in the past, and ITO RRAM devices also exhibit stable, excellent switching characteristics and high switching ratio memory windows [24,25,26].

Seo et al. discussed transparent nonvolatile resistive random access memory (Nv-T-RRAM) devices using a ZnO material and indium tin oxide (ITO) film electrodes. The ITO/ZnO/ITO structure exhibits a uni-polar electrical switching conduction behavior. T-RRAM devices may have future applications in biotechnology and high-switching-speed logic operations. Transparent nonvolatile resistive random access memory (Nv-T-RRAM) devices also show great potential in AI technology applications due to their memory array structure, cross-array format style, memory computation techniques, and low power cost [12,27]. In previous studies, indium tin oxide (ITO) materials were candidates for top/bottom transparent electrode materials because of their thermal stability, high bandgap properties, good transmission properties, and excellent conduction properties [12,13,14,15]. In order to realize the transparent nonvolatile resistive random access memory (Nv-T-RRAM) devices in this study, we chose ITO_X_:SiO_2_ films with low cost and a long continuous processing time in the same RF sputtering vacuum system. In addition, the transparency, high conductivity, electrical conduction mechanism, switching characteristics, and electrical conduction transfer mechanism characteristics of transparent ITO/ITO_X_/ITO RRAM devices make them important candidates for nonvolatile resistive random access memory device applications. In addition, the influence of different oxygen process gas deposition parameters on transparent ITO/ITO_X_/ITO-structured RRAM devices is discussed.

In our previous study, the electrical current intensity was the main reason for the soft breakdown processing behavior of the switching resistive thin film layer. The behavior of the bipolar resistance switching properties for the different compliance currents of Zn:SiO_2_ thin film RRAM devices were thoroughly analyzed. 

Due to the low compliance current there is lower possibility of the metal precipitation becoming larger. 

According to the Arrhenius equation, the activation energy for the current compliance rose from 10 μA to 100 μA. In the conductance current curve fitting, the HRS/LRS states at different operating compliance currents exhibited a hopping conduction mechanism. In addition, the dielectric film is prone to dense rupture under the action of a conduction current, and the denser metal ions accumulate to form conductive filaments. For different increases in current compliance, shorter jump distances were obtained by a rise in the diameter of the metal precipitates and the jump conduction behavior of the carriers. Finally, the hopping distance value of thin film RRAM devices was caused by the larger diameter metal ions forming the initial metal filament path when the different compliance current parameters were increased [3,28,29,30].

In this study, the ITO_X_:SiO_2_ thin film was prepared by using a co-sputtering deposition technology on a TiN/Si substrate to form the RRAM devices. Aluminum (Al) electrode materials were prepared using metal masks and thermal evaporation techniques to form a metal–insulator–metal (MIM) Al/ITO_X_:SiO_2_/TiN/Si RRAM structure. To explore the resistive switching mechanism of ITO_X_:SiO_2_ thin film RRAM devices at the interface of the TiN electrode and the ITO_X_:SiO_2_ thin film, the Al/ITO_X_:SiO_2_/TiN structure device was processed in this study. In addition, *I-V* curves of RRAM devices with temperature-dependent conductivity mechanisms were determined to study the resistive switching behavior.

## 2. Experimental Detail

In this study, ITO_X_:SiO_2_ thin films were deposited using a co-sputtering technique (fabrication parameters: RF power of 60 W for ITO and 100 W for SiO_2_, chamber pressure of 20 mTorr, sputtering time of 10 min). In addition, ITO_X_:SiO_2_ thin films were prepared on the TiN/Si substrate for an argon (Ar) concentration of 10 sccm and 0, 5, and 10 sccm oxygen (O_2_) gas, respectively. The X-ray diffraction (XRD) patterns of the crystalline phase of ITO_X_:SiO_2_ thin films were obtained at 2θ degree ranging from 20° to 60° using the Bruker D8 multifunction high power mechanism. The microstructure and surface morphologies were observed using TESCAN GAIA3, dual beam (focused ion beam and electron (FIB)).

**Figure 1:**
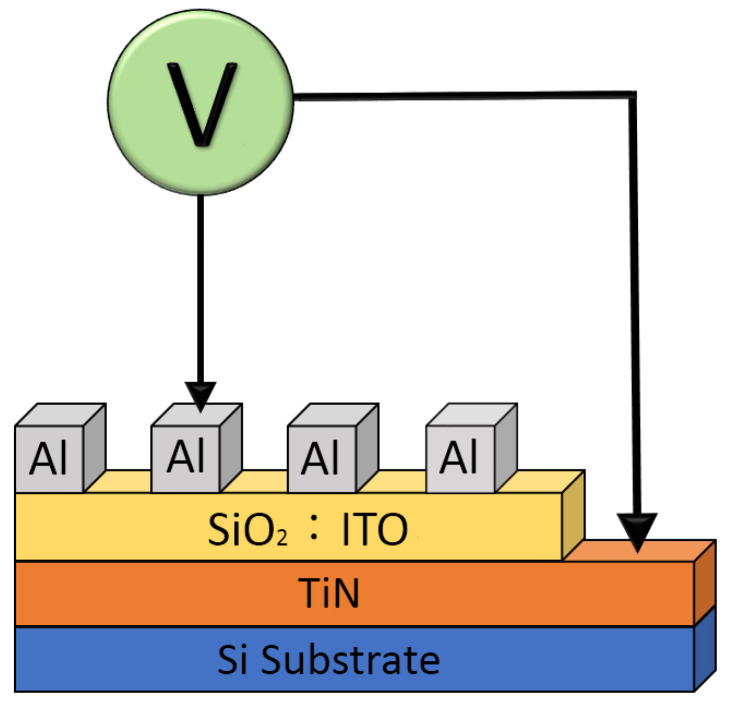
The metal–insulator–metal (MIM) structure of the Al/ITO_X_:SiO_2_/TiN/Si RRAM device.

In Figure 1, the aluminum (Al) top electrodes (100 nm thickness and 0.05 cm diameter) of the Al/ITO_X_:SiO_2_/TiN/Si (MIM) structure RRAM device was fabricated using the E-beam evaporation technology at 5 × 10^−6^ torr. Electrical current versus applied voltage (*I–V*) curves properties of the Al/ITO_X_:SiO_2_/TiN/Si structure RRAM devices were determined by using the semiconductor parameter analyzer (HP4156C) mechanism. In addition, the endurance and resistance switching properties were measured. The electrical current conduction theory for SET and RESET states were also examined. 

## 3. Results and Discussion

**Figure 2:**
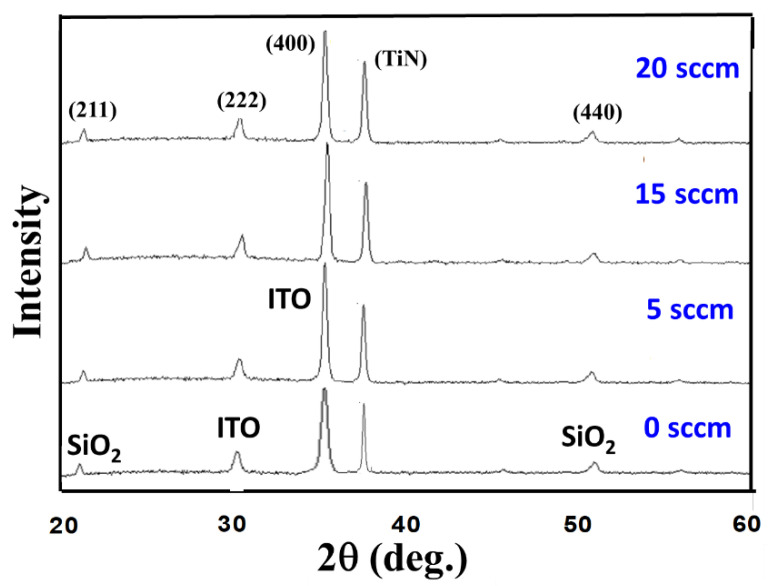
X-ray diffraction patterns of the ITO_X_:SiO_2_ thin films at different oxygen gas flows.

**Figure 3:**
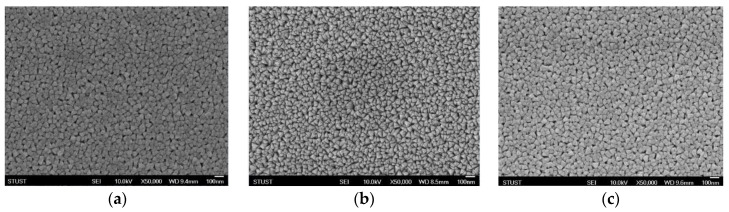
The surface morphology of ITO_X_:SiO_2_ thin films at oxygen gas flows of (**a**) 0 sccm, (**b**) 5 sccm, and (**c**) 10 sccm.

Figure 2 shows the XRD patterns of the ITO_X_:SiO_2_ thin films prepared at different oxygen gas flow rates with 60 W and 100 W RF power for the ITO target and SiO_2_ target, respectively. The (222) and (400) ITO, SiO_2_, and TiN peaks of ITO_X_:SiO_2_ thin films were found. All ITO_X_:SiO_2_ thin films showed the preferred (400) phase orientation of 35°. In addition, the (211) SiO_2_ phase orientation of thin film at 2θ degree of 22° was also observed. From the XRD patterns, the ITO textured orientations of the ITO_X_:SiO_2_ thin films for the different oxygen gas flows exhibited peak intensities at about 35°. In XRD results, the preferred peak (400) phase of ITO_X_:SiO_2_ thin film for O_2_ = 5 sccm was selected from the procedure parameters of the experimental details. The full width at half maximum (FWHM) of the ITO_X_:SiO_2_ thin film was calculated as 0.2, the grain sizes of the surface morphology were about 1–2 nm, as shown in Figure 3.

Figure 3 presents the surface structure and images of ITO_X_:SiO_2_ thin films for different oxygen gas flows of 0, 5, and 10 sccm. In Figure 3b,c, more pores are found for 5–10 sccm specimens, and a denser surface is found in Figure 3a. In addition, the film surface of the 5 sccm oxygen specimens showed a uniform and flat structure. In general, oxygen vacancies in thin films contribute to the current transport paths of various oxygen species in the co-sputtering fabrication process. Oxygen gas reacts with ITO and SiO_2_ target materials and oxygen ions fill up oxygen vacancies in the thin film. The electrical current transmission paths for the variation in the x value in the ITO_X_:SiO_2_ thin film composition were observed. However, excess oxygen ions filling the vacancies led to the formation of defects, and the reduction in the current transport path affected the insulating properties of the ITOX:SiO_2_ thin film.

**Figure 4:**
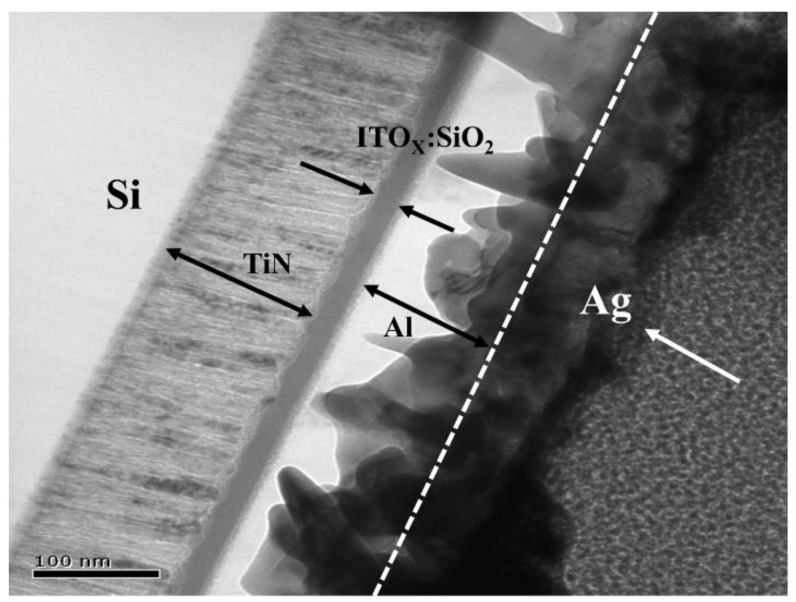
Cross-sectional morphology of the ITO_X_:SiO_2_ thin film RRAM device.

According to the cross-section morphology of the SEM images, the Al/ITO_X_:SiO_2_/TiN structure device was observed and found for every layer. The thickness of the aluminum (Al) top electrodes and TiN bottom electrodes were about 100 nm. In addition, the thickness of the ITO_X_:SiO_2_ thin films was about 30 nm, as shown in Figure 4. In addition, Ag/Pt conductive metal materials were used for the FIB TEM mechanism, and the Ag material melted on the Al/ITO_x_:SiO_2_/TiN structures at high-temperature measurements. For the Al/ITO_X_:SiO_2_/TiN/Si (MIM) structure in this study, the ITO_X_:SiO_2_ thin film RRAM devices were prepared using a co-sputtering technology. Based on previous studies, Ag/SiO_2_/ITO structures with intrinsic threshold switching and bipolar memory switching have been reported and investigated. In our experimental details and results, the composite material and sharp interface of the Al/ITO_X_:SiO_2_/TiN/Si (MIM) structure could not be observed or found [3,27,28,29].

**Figure 5:**
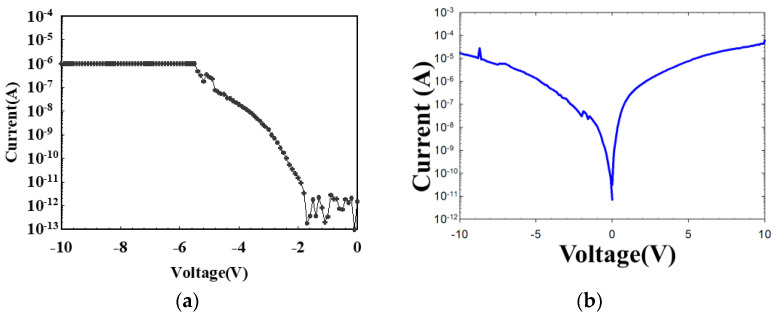
The *I-V* curves of (**a**) the ITO_X_:SiO_2_ thin film RRAM device and (**b**) the un-doped SiO_2_-based thin film during the initial electrical forming process.

The electroforming procedure of the RRAM devices displayed the soft-breakdown effect and low resistance state (LRS) of the RRAM devices above the threshold voltage. The authors believe there are different filament electroforming effects for different RRAM devices. In our previous study, the thin films prepared using different oxygen gas parameters exhibited different *I-V* curve properties [27,28]. In addition, the typical *I-V* curve properties of RRAM devices were also affected by different constant compliance current parameters. In this study, the electroforming procedure was used, and the RRAM devices showed typical *I-V* curves. In this study, the electrical current compliance was 10^−6^ mA, as depicted in Figure 5a. In addition, the I-V curves of the un-doped SiO_2_-based thin film RRAM device and the as-deposited SiO_2_ thin film are shown in Figure 5b. The *I-V* curve results indicated that the un-doped SiO_2_ thin film exhibited a volatile memory effect.

**Figure 6:**
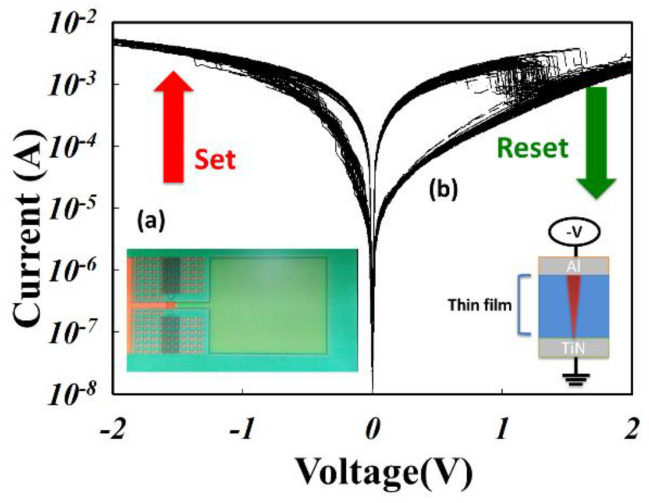
Typical I-V switching property showing the LRS/HRS states of the ITO_X_:SiO_2_ RRAM device measured with 100 repetitions. Inset shows (**a**) the real device image and (**b**) the MIM structure.

In Figure 6, the *I-V* curve characteristics of the ITO_X_:SiO_2_ thin-film RRAM device show that when the applied negative voltage was higher than the set voltage, the current started to decrease and the RRAM device was switched from HRS to LRS (called the SET process). The electrical current turned back to the HRS state when the applied voltage became positive and higher than the reset voltage (called the RESET process). To investigate the *I–V* curves’ switching effect, the LRS and HRS states of the ITO_X_:SiO_2_ RRAM devices were measured 100 times, as depicted in Figure 6. In Figure 6a, the real image of the ITO_X_:SiO_2_ thin film RRAM devices is shown. The set voltage was decreased gradually, and the reset state showed typical *I–V* switching properties when the Al/ITO_X_:SiO_2_/TiN/Si RRAM devices were measured 100 times. 

**Figure 7:**
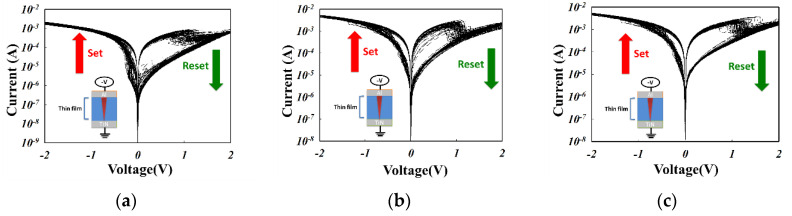
The typical characteristics of the I-V curves of the Al/ITO_X_:SiO_2_/TiN/Si RRAM device at (**a**) 0 sccm, (**b**) 5 sccm, and (**c**) 10 sccm oxygen (with 100 repetitions).

In Figure 7, the oxygen gas flow during preparation of the ITO_X_:SiO_2_ thin film increased from 0 to 10 sccm gradually. The ITO_X_:SiO_2_ thin film RRAM device exhibited more typical bipolar switching properties between the set/reset states with 0, 5, and 10 sccm oxygen. Furthermore, both set/reset processes exhibited less than 2.5 V operating voltage and good memory switching properties. The oxygen vacancies and defects of the ITO_X_:SiO_2_ thin films might be repaired and filled up by oxygen in the co-sputtering deposition process.

**Figure 8:**
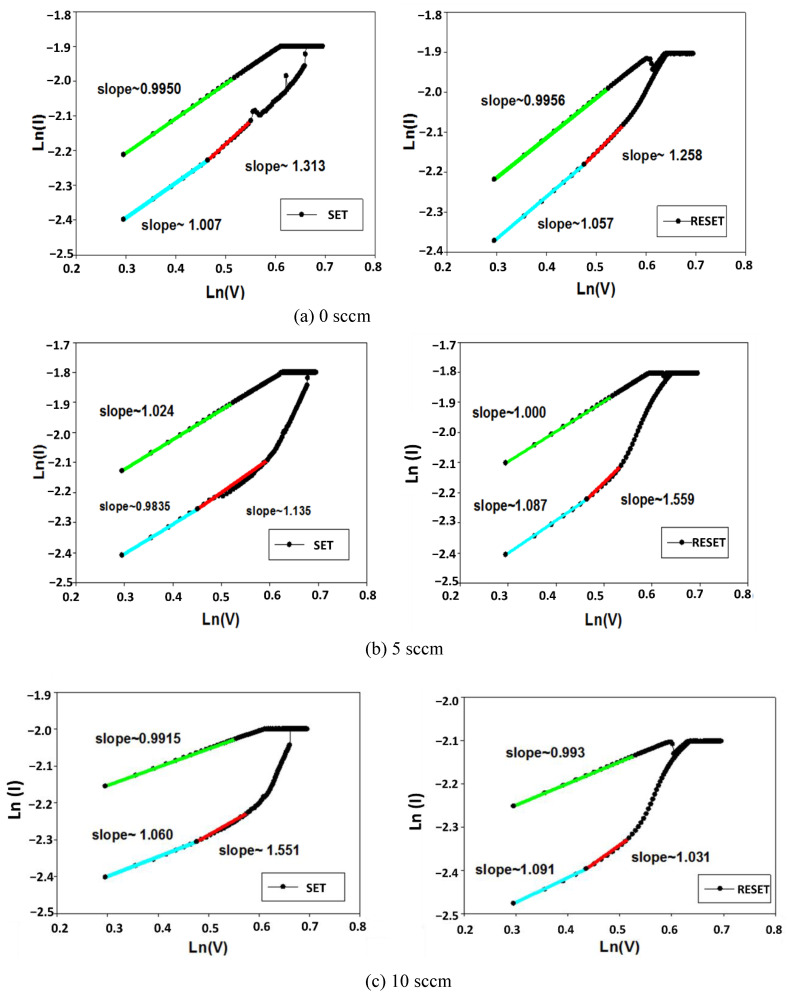
Ln(I)-Ln(V) plots of Al/ITO_X_:SiO_2_/TiN/Si RRAM with (**a**) 0 sccm, (**b**) 5 sccm, and (**c**) 10 sccm oxygen.

Recently, many different electrical conduction transport models and mechanisms of thin film RRAM devices, such as the Schottky barrier height caused by the trapped charge effect, the conduction filament path formation, the oxygen vacancy effect, and the carrier tunneling effect, have been used to explain the conduction model of thin film RRAM devices [31,32,33,34,35]. The resistive switching characteristics of ITO_X_:SiO_2_ thin-film RRAM devices were investigated by analyzing the *I-V* curve results of the ITO_X_:SiO_2_ thin-film RRAM devices. In addition, the electrical transport conduction mechanisms of the thin-film RRAM devices include the ohmic transport mechanism and the space charge limited conductance (SCLC) transport conduction mechanism. In addition, the hopping conduction mechanism under low applied voltages has been widely discussed. In Figure 8, the Ln(I)-Ln(V) curve characteristics of Al/ITO_X_:SiO_2_/TiN/Si RRAM devices are shown and fitted for an ITO target with 60 W RF power and a SiO_2_ target with 100 W RF power at different oxygen flow conditions of 0, 5 and 10 sccm, respectively. 

In Figure 8b,c, the I-V curves of the films in 5 and 10 sccm oxygen show a clear bipolar switching behavior, and the 0 sccm specimen exhibits a large resistive ON/OFF ratio in the HRS and LRS states. The current conduction mechanism of the thin-film RRAM device is ohmic conduction (the slope is about 1 when the external electric field is dominant), and the hopping conduction mechanism of the SET and RESET states at low operating voltages was observed and investigated (blue and green lines in Figure 8). However, the electrical transport mechanism for current reduction was caused by the defects filled from the carriers by high operating voltage. The conduction mechanisms exhibited the SCLC electrical transport mechanism (slope was about 2).

For the operating voltage applied (1 < slope < 2), the current densities of Ohmic conduction and SCLC were expressed as

(1) $$J=nq\mu E\exp(\frac{-\Delta E_{ac}}{kT})$$

(2) $$J=\frac{9\varepsilon_{i}\mu V^{2}}{8d^{3}}$$

where the variables represent current density (J), electron concentration (n), electron mobility (μ), electric field (E), activation energy (ΔE_ac_), Boltzmann’s constant (k), the dynamic permittivity of the insulator layer (ε_i_), the thickness of the insulator layer (d), and the applied voltage (V).

To investigate the electrical conduction behavior of the initial metallic filament forming conduction, the hopping conduction mechanism and the Schottky emission conduction were calculated using ln*I–V* and ln*I–V^1/2^* curves. For the Schottky emission conduction equation,

(3) $$J=A^{*}T^{2}\exp\left[-q\left(\Phi_{B}-\sqrt{\frac{qE_{i}}{4\pi \varepsilon_{i}}}\right)/\mathrm{kT}\right]$$

where the variables represent the absolute temperature (T), the Schottky barrier height (Φ*_B_*), the insulator permittivity (*ε_i_*), Boltzmann’s constant (k), and Richardson constant (A*). 

To calculate the $\ln\left(\frac{I}{T^{2}}\right)-\sqrt{V}$ curve, the Schottky conduction equation of the RRAM devices was transformed to the *I-V* curve fitting. 

For the hopping conduction,

(4) $$J=qN_{a}V_{o}e^{\frac{-q\Phi_{T}}{kT}}e^{\frac{qaV}{2dkT}}$$

where *d*, Φ*_T_*, *v*_0_, *N*, and *a* are the film thickness, the barrier height of hopping, the intrinsic vibration frequency, the density of space charge, and the mean hopping distance, respectively [12].

**Figure 9:**
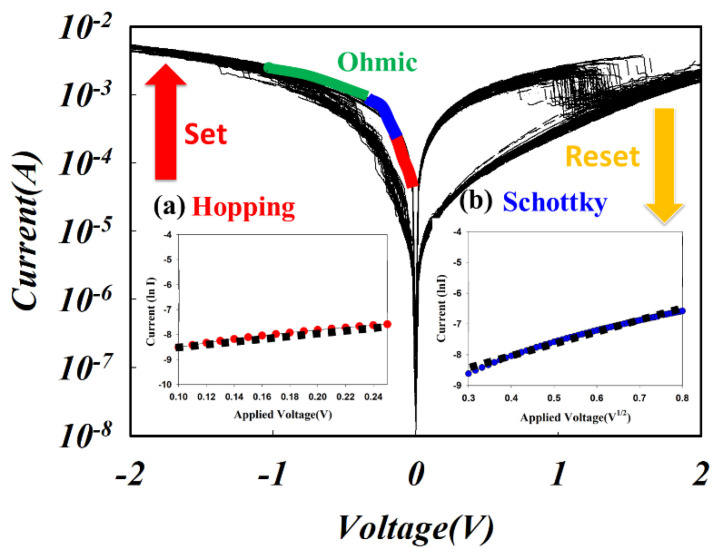
The *I-V* curves of Al/ITO_X_:SiO_2_/TiN/Si RRAM devices under 5 sccm oxygen gas for (**a**) Ln(I) versus (V) and (**b**) Ln(I) versus (V^1/2^) curve fitting.

In Figure 9, the electrical transfer mechanism of the 5 sccm ITO_X_:SiO_2_ thin film RRAM devices at high voltage exhibited the Schottky emission mechanisms as judged by the ln*I–V^1/2^* curve fittings. In addition, the hopping conduction model of the thin films from the RRAM devices can be observed from the ln*I–V* curve. As shown in Figure 9, the Schottky emission mechanism was caused by the shallowly trapped electrons jumping the activation energy barrier, causing the low electrical conduction current [12]. From the above experimental results, the oxygen gas procedure parameters are an important factor affecting the resistive switching operations of thin film RRAM devices [2,3,4,5,6,7,8,9,10,11,12]. In the electrical filament path of thin film RRAM devices, the formation and rupture process of the electrical conducting filaments of the switching mechanisms was found. The *I-V* curve switching properties of the ITO_X_:SiO_2_ thin film RRAM devices were affected by many oxygen vacancies that existed in the interface between the electrode and ITO_X_:SiO_2_ thin film.

**Figure 10:**
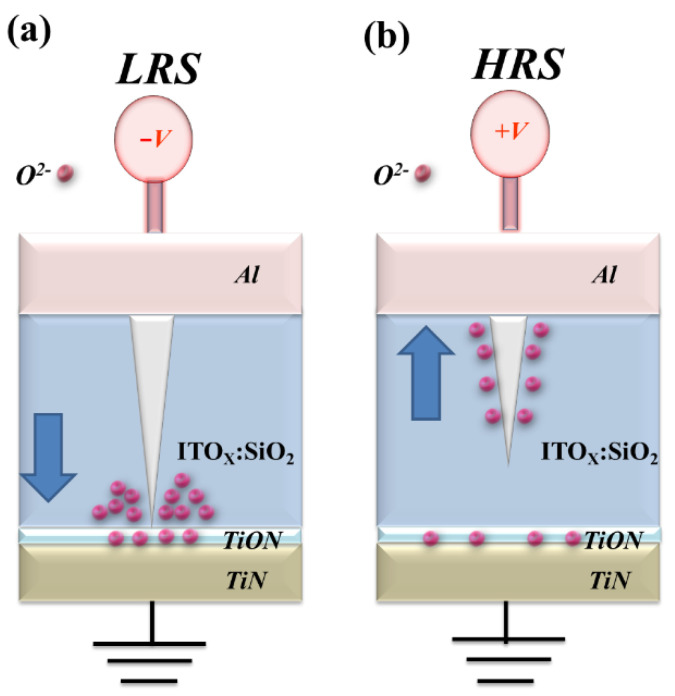
The metallic filament physical path model of the ITO_X_:SiO_2_ thin film RRAM device for (**a**) LRS (set) and (**b**) HRS (reset) state.

Figure 10 presents the metallic filament path model for the LRS and HRS states of the ITO_X_:SiO_2_ RRAM device. Figure 10a illustrates the metallic filament path diagram of the ITO_X_:SiO_2_ thin film RRAM device with applied bias above the set state. The oxygen vacancies exist in the interface region between the TiN bottom and the thin film of the ITO_X_:SiO_2_ RRAM device and gradually accumulate at the LRS states. Figure 10b shows the continuing oxidation reaction of the thin electrical metal metallic filament path at high positive voltage. The thin metal metallic filament becomes affected by oxygen atoms, closing off the bottom electrode area. Finally, from the *I-V* curves, the bipolar switching properties of the ITO_X_:SiO_2_ RRAM device were determined by utilizing the physical forming transfer model in the top and bottom electrodes [1,2,3,4,5,6,7,8,9,10,11,12].

**Figure 11:**
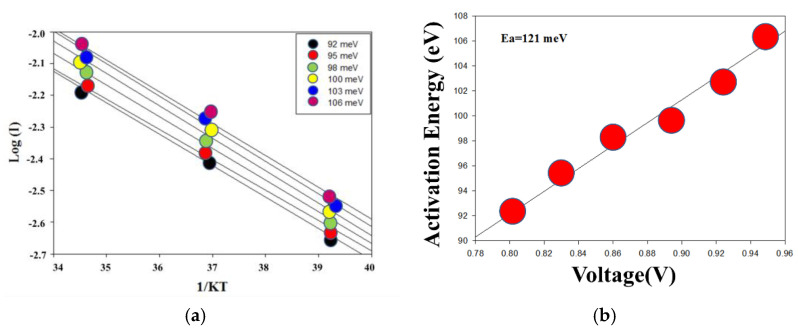
The (**a**) (–lnI) versus (1/kT) curve and (**b**) activation energy versus applied voltage (V) of the hopping conduction theory in an Arrhenius plot equation for the ITO_X_:SiO_2_ thin film RRAM device.

In order to calculate the activation energy of the ITO_X_:SiO_2_ thin film RRAM devices under the hopping conduction theory, the (*–lnI*) versus (*1/kT*) curves of the Arrhenius plot equation were determined as shown in Figure 11a,b. The ln(I)-1/kT curves of ITO_X_:SiO_2_ thin film RRAM devices for various current compliances were measured at different temperatures. The activation energy extraction was determined to be 121 meV. In previous studies, the equation of the hopping conduction mechanism was

$$E_{A,exp}=-\frac{\partial \log I}{\partial \left(1\frac{1}{kT}\right)}=E_{C}-E_{F}-qV_{A}\frac{\Delta z}{2u_{a}}$$

by simplifying the activation energy barrier equation, where the variables represent the active energy (E_a_), the applied voltage (k), the average inter-trap distance (∆z), and the thickness (u_a_). In addition, the average inter-trap distance (∆z) of ITO_X_:SiO_2_ thin film RRAM devices was calculated to be about 6.5 nm [2,3,4,5,6,7,8,9,10,11]. According to our previous study, the average hopping distance of the Zn:SiO_2_ thin film RRAM devices was about 1.44 nm. In these experimental results, the hopping distance was determined from the different compliance current, and activation energy of the RRAM devices [36].

**Figure 12:**
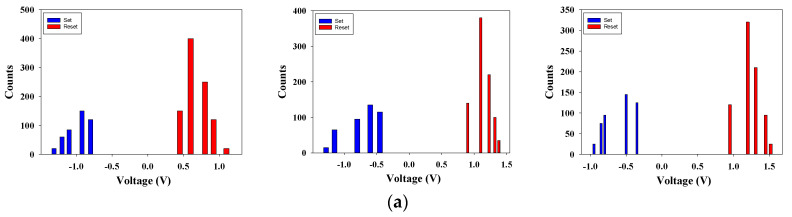

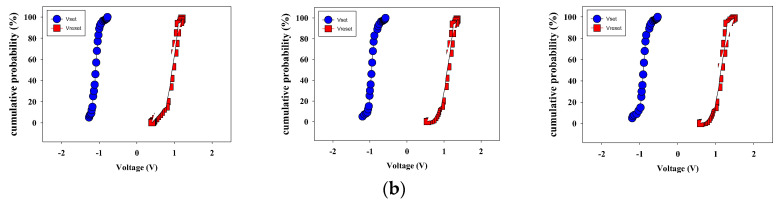
(**a**) The count distribution and (**b**) the cumulative probability results of the RRAM devices (400 repetitions).

Figure 12 shows the statistical distribution results for set and reset state voltages of the ITO_X_:SiO_2_ RRAM device from 400 measurements, respectively. In determining the count distribution values of Figure 12a, the set/reset voltage was measured with an applied voltage of −1.2 to 1.5 V. Figure 12b presents the cumulative probability statistical results for the *I-V* curve switching properties of the ITO_X_:SiO_2_ thin film RRAM device for set and reset states. The above results indicate that the set/reset voltage values of the ITO_X_:SiO_2_ thin film RRAM device shifted and decreased due to the effect of excess oxygen during the deposition process.

**Figure 13:**
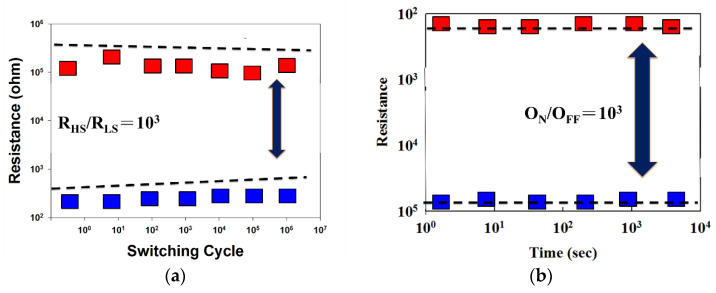
Switching endurance results of the ITO_X_:SiO_2_ thin film RRAM device showing (**a**) retention properties and (**b**) endurance properties.

From the above experimental results, the ITO_X_:SiO_2_ thin film RRAM exhibited optimal properties and switching endurance characteristics of the Al/ITO_X_:SiO_2_/TiN/Si RRAM device with 5 sccm oxygen gas, as shown in Figure 13a. The 5 sccm RRAM devices exhibited a low set/reset voltage over one million switching cycles. The resistance versus switching time between the HRS and LRS states was obviously observed. In addition, the ITO_X_:SiO_2_ thin film RRAM device displayed a typical bipolar switching behavior and a greater than 10^3^ HRS/LRS memory ratio. Figure 13b demonstrates that the RRAM device showed no significant changes for the O_N_/O_FF_ ratio switching behavior cycling versus the time curves for more than 10^3^ s in the extrapolation calculation anticipation.

Table 1 compares electrical parameters for doped SiO_2_-based RRAM structures. Zn:SiO_2_, Ni:SiO_2_, Sn:SiO_2_, and Gd:SiO_2_ RRAM devices all exhibited good operation current stability properties. The SiO_2_-based RRAM device has an operation current of approximately 1 × 10^−2^ to 5 × 10^−4^ A and set and reset voltages of approximately 1 V and 2 V, respectively. According to our previous study, the device size might not impact the overall device performance. The device size of thin film RRAM devices was reported as an important factor for the device performance and initial metal filament processing of hopping, Schottky emission, and SCLC conduction mechanism [37]. The endurance and retention ratios of thin film RRAM devices were about 10^6^ and 10^9^, respectively [1,2,3,4,5,6,7,8,9,10,11,12,31,32,33,34]. However, the ITO:SiO_2_ RRAM devices exhibit typical endurance and retention properties. In addition, as shown in this study, the ITO:SiO_2_ RRAM device has a low set/reset voltage, which reduces losses in consumer electronic devices.

## 4. Conclusions

In this study, the resistance switching behavior of Al/ITO_X_:SiO_2_/TiN/Si RRAMs was investigated and demonstrated. Compared to the oxygen-free specimen (O_2_ = 0 sccm), the 5 sccm and 10 sccm specimens showed better switching and HRS/LRS resistance ratio characteristics. In addition, the ITO_X_:SiO_2_ thin film RRAM devices exhibited typical bipolar behavior with an on/off memory window ratio of 10^3^ and switching cycle reliability of 10^4^ under an RF power of 60 W for ITO and 100 W for SiO_2_ and 10 sccm oxygen gas.

The conduction mechanism of RRAM devices exhibits ohmic conduction and hopping conduction behavior in the SET/RESET state. In addition, the conductive mechanism partially exhibits SCLC behavior when a high operating voltage is applied with an activation energy extraction of 121 meV. Finally, the count distribution results show that the set/reset voltage is −1.2 to 1.5 V. The typical *I-V* curve switching properties suggest that these Al/ITO_X_:SiO_2_/TiN/Si structure RRAM devices are suitable for next-generation nonvolatile memory devices. 

## Figures and Tables

**Table 1:** Comparative table of SiO_2_ RRAM structures with different electrical parameters [1,2,3,4,5,6,7,8,9,10,11,12,38,38,39].

	Zn:SiO_2_ [34]	Ni:SiO_2_ [34]	Sn:SiO_2_ [32]	Gd:SiO_2_ [31]	ITO:SiO_2_
Operation current	1 × 10^−3^ A	1 × 10^−3^ A	1 × 10^−2^ A	5 ×10^−4^ A	5 × 10^−3^ A
Operation stability	Good	Good	Good	Good	Good
Set voltage	<1 V	<1.5 V	<1 V	<0.5 V	<0.5 V
Reset voltage	<1.5 V	<2 V	<1.5 V	<1 V	<1 V
Endurance	>10^7^	>10^6^	>10^7^	>10^6^	>10^6^
Retention	>10^9^	>10^9^	>10^7^	>10^6^	>10^6^

## Data Availability

Not applicable.

## References

[1] Li L., Dai T.J., Liu K., Chang K.C., Zhang R., Lin X., Liu H.J., Lai Y.C., Kuo T.P. (2021). Achieving complementary resistive switching and multi-bit storage goals by modulating the dual-ion reaction through supercritical fluid-assisted ammoniation. Nanoscale.

[2] Li L., Chang K.C., Zhang R., Lin X., Lai Y.C., Kuo T.P. (2020). Variable-temperature activation energy extraction to clarify the physical and chemical mechanisms of the resistive switching process. Nanoscale.

[3] Li L., Chang K.C., Ye C., Lin X., Zhang R., Xu Z., Xiong W., Zhou Y., Kuo T.P. (2020). An Indirect Way to Achieve Comprehensive Performance Improvement of Resistive Memory: When Hafnium Meets ITO in Electrode. Nanoscale.

[4] Chang K.C., Dai T.J., Li L., Lin X.N., Zhang S.D., Lai Y.C., Liu H.J., Syu Y.E. (2020). Unveiling the influence of surrounding materials and realization of multi-level storage in resistive switching memory. Nanoscale.

[5] Chang K.C., Zhang R., Chang T.C., Tsai T.M., Chu T.J., Chen H.L., Shih C.C., Pan C.H., Su Y.T., Wu P.J. High performance, excellent reliability multifunctional graphene oxide doped memristor achieved by self-protective compliance current structure. Proceedings of the IEEE International Electron Devices Meeting (IEDM).

[6] Ye C., Xu Z., Chang K.C., Li L., Lin X.N., Zhang R., Zhou Y., Xiong W., Kuo T.P. (2019). Hafnium nanocrystals observed in a HfTiO compound film bring about excellent performance of flexible selectors in memory integration. Nanoscale.

[7] Lanza M., Waser R., Ielmini D., Yang J.J., Goux L., Suñe J., Kenyon A., Mehonic A., Spiga S., Rana V. (2021). Standards for the Characterization of Endurance in Resistive Switching Devices. ACS Nano.

[8] Sebastian A., Le Gallo M., Khaddam-Aljameh R., Eleftheriou E. (2020). Memory devices and applications for in-memory computing. Nat. Nanotechnol..

[9] Sebastian A., Le Gallo M., Khaddam-Aljameh R., Funck C., Menzel S. (2021). Comprehensive model of electron conduction in oxide-based memristive devices. ACS Appl. Electron. Mater..

[10] Dalgaty T., Castellani N., Turck C., Harabi K.E., Querlioz D., Vianello E. (2021). In situ learning using intrinsic memristor variability via Markov chain Monte Carlo sampling. Nat. Electron..

[11] Lanza M., Wong H.-S.P., Pop E., Ielmini D., Strukov D., Regan B., Larcher L., Villena M., Yang J., Goux L. (2019). Recommended methods to study resistive switching devices. Adv. Electron. Mater..

[12] Chen K.-H., Cheng C.-M., Wang N.-F., Hung H.-W., Li C.-Y., Wu S. (2023). First Order Rate Law Analysis for Reset State in Vanadium Oxide Thin Film Resistive Random Access Memory Devices. Nanomaterials.

[13] Rahaman S., Maikap S. (2010). Low power resistive switching memory using Cu metallic filament in Ge0.2Se0.8 solid-electrolyte. Microelectron. Reliab..

[14] Choi S.J., Lee J.H., Bae H.J., Yang W.Y., Kim T.W., Kim K.H. (2008). Improvement of CBRAM resistance window by scaling down electrode size in pure-GeTe film. IEEE Electron Device Lett..

[15] Goux L., Opsomer K., Degraeve R., Müller R., Detavernier C., Wouters D.J., Jurczak M., Altimime L., Kittl J.A. (2011). Influence of the Cu-Te composition and microstructure on the resistive switching of Cu-Te/Al_2_O_3_/Si cells. Appl. Phys. Lett..

[16] Bernard Y., Gonon P., Jousseaume V. (2010). Resistance switching of Cu/SiO_2_ memory cells studied under voltage and current-driven modes. Appl. Phys. Lett..

[17] Tsuji Y., Sakamoto T., Banno N., Hada H., Aono M. (2010). Off-state and turn-on characteristics of solid electrolyte switch. Appl. Phys. Lett..

[18] Tsunoda K., Fukuzumi Y., Jameson J.R., Wang Z., Griffin P.B., Nishi Y. (2007). Bipolar resistive switching in polycrystalline TiO_2_ films. Appl. Phys. Lett..

[19] Choi H., Pyun M., Kim T.-W., Hasan M., Dong R., Lee J., Park J.-B., Yoon J., Seong D.-J., Lee T. (2009). Nanoscale Resistive Switching of a Copper–Carbon-Mixed Layer for Nonvolatile Memory Applications. IEEE Electron. Device Lett..

[20] Jo S.H., Kim K.H., Lu W. (2009). High-density crossbar arrays based on a Si memristive system. Nano Lett..

[21] Yao J., Sun Z., Zhong L., Natelson D., Tour J.M. (2010). Resistive Switches and Memories from Silicon Oxide. Nano Lett..

[22] Tsai T.-M., Chang K.-C., Chang T.-C., Zhang R., Wang T., Pan C.-H., Chen K.-H., Chen H.-M., Chen M.-C., Tseng Y.-T. (2016). Resistive Switching Mechanism of Oxygen-Rich Indium Tin Oxide Resistance Random Access Memory. IEEE Electron. Device Lett..

[23] Zhang R., Young T.-F., Chen M.-C., Chen H.-L., Liang S.-P., Syu Y.-E., Sze S.M., Chang K.-C., Chang T.-C., Tsai T.-M. (2014). Characterization of Oxygen Accumulation in Indium-Tin-Oxide for Resistance Random Access Memory. IEEE Electron. Device Lett..

[24] Lin C.-Y., Chang K.-C., Chang T.-C., Tsai T.-M., Pan C.-H., Zhang R., Liu K.-H., Chen H.-M., Tseng Y.-T., Hung Y.-C. (2015). Effects of Varied Negative Stop Voltages on Current Self-Compliance in Indium Tin Oxide Resistance Random Access Memory. IEEE Electron. Device Lett..

[25] Fujimoto M., Koyama H. (2006). TiO_2_ anatase nanolayer on TiN thin film exhibiting high-speed bipolar resistive switching. Appl. Phys. Lett..

[26] Seo S., Lee M.J., Seo D.H., Jeoung E.J., Suh D.-S., Joung Y.S., Yoo I.K., Hwang I.R., Kim S.H., Byun I.S. (2004). Reproducible resistance switching in polycrystalline NiO films. Appl. Phys. Lett..

[27] Donley C., Dunphy D., Paine D., Carter C., Nebesny K., Lee P., Alloway D., Armstrong N.R. (2001). Characterization of Indium−Tin Oxide Interfaces Using X-ray Photoelectron Spectroscopy and Redox Processes of a Chemisorbed Probe Molecule: Effect of Surface Pretreatment Conditions. Langmuir.

[28] Chen K.-H., Kao M.-C., Huang S.-J., Li J.-Z. (2017). Bipolar Switching Properties of Neodymium Oxide RRAM Devices Using by a Low Temperature Improvement Method. Materials.

[29] Li Z., Böruer P.D., Schmidt H., Bolívar P.H., Choubey B. Bidirectional Transition between Threshold and Bipolar Switching in Ag/SiO2/ITO Memristors. Proceedings of the 2022 IEEE 22nd International Conference on Nanotechnology (NANO).

[30] Chen K.H., Cheng C.M., Li C.Y., Huang S.J. (2018). Hopping conduction distance of bipolar switching GdOx resistance random access memory thin films devices modified by different constant compliance current. Microelectron. Reliab..

[31] Kim D.C., Lee M.J., Ahn S.E., Seo S., Park J.C., Yoo I.K., Baek I.G., Yim E.K., Lee J.E., Park S.O. (2006). Improvement of resistive memory switching in NiO using IrO_2_. Appl. Phys. Lett..

[32] Oka T., Nagaosa N. (2005). Interfaces of Correlated Electron Systems: Proposed Mechanism for Colossal Electroresistance. Phys. Rev. Lett..

[33] Choi B.J., Jeong D.S., Kim S.K., Rohde C., Choi S., Oh J.H., Kim H.J., Hwang C.S., Szot K., Waser R. (2005). Resistive switching mechanism of TiO2 thin films grown by atomic-layer deposition. J. Appl. Phys..

[34] Jeon S.H., Park B.H., Lee J., Lee B., Han S. (2006). First-principles modeling of resistance switching in perovskite oxide material. Appl. Phys. Lett..

[35] Rozenberg M.J., Inoue I.H., Sánchez M.J. (2006). Strong electron correlation effects in nonvolatile electronic memory devices. Appl. Phys. Lett..

[36] Chen K.H., Zhang R., Chang T.C., Tsai T.M., Chang K.C., Lou J.C., Young T.F., Chen J.H., Shih C.C., Tung C.W. (2013). Hopping conduction distance dependent activation energy characteristics of Zn:SiO_2_ resistance random access memory devices. Appl. Phys. Lett..

[37] Chang K.-C., Chang T.-C., Tsai T.-M., Zhang R., Hung Y.-C., Syu Y.-E., Chang Y.-F., Chen M.-C., Chu T.-J., Chen H.-L. (2015). Physical and chemical mechanisms in oxide-based resistance random access memory. Nanoscale Res. Lett..

[38] Chen K.H., Chang K.C., Chang T.C., Tsai T.M., Liang S.P., Young T.F., Syu Y.E., Sze S.M. (2016). Improvement of Bipolar Switching Properties of Gd:SiOx RRAM Devices on Indium Tin Oxide Electrode by Low-Temperature Supercritical CO_2_ Treatment. Nanoscale Res. Lett..

[39] Chen K.H., Chang K.C., Chang K.C., Chang K.M., Sze S.M. (2016). Effect of different constant compliance current for hopping conduction distance properties of the Sn:SiOx thin film RRAM device. Appl. Phys. A.

